# Altered Adipogenesis in Zebrafish Larvae Following High Fat Diet and Chemical Exposure Is Visualised by Stimulated Raman Scattering Microscopy

**DOI:** 10.3390/ijms18040894

**Published:** 2017-04-24

**Authors:** Marjo J. den Broeder, Miriam J. B. Moester, Jorke H. Kamstra, Peter H. Cenijn, Valentina Davidoiu, Leonie M. Kamminga, Freek Ariese, Johannes F. de Boer, Juliette Legler

**Affiliations:** 1 Institute of Environmental, Health and Societies, Brunel University, UB8 3PH London, UK; marjo.denbroeder@brunel.ac.uk; 2 Institute for Environmental Studies, Vrije Universiteit Amsterdam, 1081 HV Amsterdam, The Netherlands; peter.cenijn@vu.nl; 3 Institute for Lasers, Life and Biophotonics, Department of Physics and Astronomy, Vrije Universiteit Amsterdam, 1081 HV Amsterdam, The Netherlands; m.j.b.moester@vu.nl (M.J.B.M.); v.davidoiu@vu.nl (V.D.); f.ariese@vu.nl (F.A.); jfdeboer@few.vu.nl (J.F.d.B.); 4 Department of Basic Sciences and Aquatic Medicine, Faculty of Veterinary Medicine, Norwegian University of Life Sciences, P.O. BOX 8146, Dep 0033 Oslo, Norway; jorke.kamstra@nmbu.no; 5 Rotterdam Ophthalmic Institute, Rotterdam Eye Hospital, 3011 BH Rotterdam, The Netherlands; 6 Radboud University Nijmegen, Faculty of Science, Department of Molecular Biology, Radboud Institute for Molecular Life Sciences, 6525 GA Nijmegen, The Netherlands; l.kamminga@science.ru.nl; 7 Radboud University Medical Center, Radboud Institute for Molecular Life Sciences, 6525 GA Nijmegen, The Netherlands

**Keywords:** adipocyte, zebrafish, SRS imaging, obesity, obesogen, TBT, TDCiPP, toxicology, endocrinology

## Abstract

Early life stage exposure to environmental chemicals may play a role in obesity by altering adipogenesis; however, robust in vivo methods to quantify these effects are lacking. The goal of this study was to analyze the effects of developmental exposure to chemicals on adipogenesis in the zebrafish (*Danio rerio*). We used label-free Stimulated Raman Scattering (SRS) microscopy for the first time to image zebrafish adipogenesis at 15 days post fertilization (dpf) and compared standard feed conditions (StF) to a high fat diet (HFD) or high glucose diet (HGD). We also exposed zebrafish embryos to a non-toxic concentration of tributyltin (TBT, 1 nM) or Tris(1,3-dichloroisopropyl)phosphate (TDCiPP, 0.5 µM) from 0–6 dpf and reared larvae to 15 dpf under StF. Potential molecular mechanisms of altered adipogenesis were examined by qPCR. Diet-dependent modulation of adipogenesis was observed, with HFD resulting in a threefold increase in larvae with adipocytes, compared to StF and HGD. Developmental exposure to TBT but not TDCiPP significantly increased adipocyte differentiation. The expression of adipogenic genes such as *pparda*, *lxr* and *lepa* was altered in response to HFD or chemicals. This study shows that SRS microscopy can be successfully applied to zebrafish to visualize and quantify adipogenesis, and is a powerful approach for identifying obesogenic chemicals in vivo.

## 1. Introduction

In the last decades, the number of obese and overweight adults and children has increased dramatically [1]. Childhood obesity is an important risk factor for developing obesity later in life, and obesity is known to increase the risk of other health complications, including cardiovascular disease and diabetes mellitus type II. Obesity is a complex multifactorial disease and, while it is clear that a diet high in fat and/or carbohydrates may lead to energy imbalance and weight gain, environmental factors also play a role in the onset of obesity [2,3]. Early-life exposure to environmental chemicals is an example of an environmental stressor that may increase the susceptibility of organisms to develop obesity [4]. Chemicals referred to as environmental obesogens may promote obesity by altering lipid metabolism, increasing the number and/or size of fat cells (adipocytes), or influencing fat storage in existing adipocytes [5,6]. Environmental obesogens may also disrupt energy balance by modifying mechanisms that regulate appetite and satiety [4,6]. Several classes of environmental chemicals, including pesticides, phthalates and flame retardants, have been shown in in vitro and experimental rodent studies to stimulate adipocyte differentiation (adipogenesis) [4]. However, robust in vivo methods for determining the effects of developmental exposures on adipocyte size and number in real time are lacking.

The alteration of adipogenesis by environmental obesogens is of concern because elevated adipocyte number and size early in development may affect the long term risk of obesity [7]. During embryonic development, early adipocytes differentiate from mesenchymal stem cells (MSCs) into committed preadipocytes, which then develop into mature adipocytes during terminal differentiation. The master regulator in adipogenesis is the peroxisome proliferator-activated receptor gamma (PPARG), and by heterodimerisation to retinoid X receptor (RXR), the PPARG:RXR dimer regulates transcription of downstream target genes [8]. Other important adipogenic factors are the CCAAT/enhancer-binding proteins (C/EBPs), Kruppel-like factors (KLFs) and signal transducers and activators of transcription (STATs), which facilitate adipocyte differentiation. There are also anti-adipogenic regulators of adipocyte differentiation identified including Wingless-type MMTV integration site family (WNT) members and GATA binding proteins (GATA) 2/3 [9,10]. Adipocytes function as the site for energy storage and are endocrine cells that secrete various hormones and cytokines necessary for homeostasis and lipid metabolism [11,12]. In humans and rodents, there are two main types of adipose tissue, white adipose tissue (WAT) and brown adipose tissue (BAT), which are derived from distinct cell lineages. Recently, a third type has been identified, the so-called beige or brite adipose tissue. WAT is the most common type of adipose tissue and serves as energy storage in the form of triglycerides, with important functions in regulating insulin sensitivity and lipid metabolism. BAT is essential for thermogenesis, while the function of beige/brite cells is less clear [13,14].

In this study, we analyzed the effects of developmental exposure to environmental obesogens on adipogenesis in the zebrafish (*Danio rerio*). We selected the zebrafish because it is a well-known model organism to study human disease and it has many advantages compared to rodents, including rapid development, a short life cycle and the availability of genome sequence information and genome editing techniques [15,16,17]. Zebrafish are an emerging model to study obesity, as lipid metabolism pathways are conserved between mammals and fish [18,19] and significant homology exists in PPAR-mediated pathways [19]. In contrast to mammals, however, zebrafish are poikilothermic and BAT and beige/brite cells have not been identified [20]. During the first days of development, zebrafish embryos are dependent on nutrients provided by the yolk sac. After the yolk sac is almost depleted at about six days post fertilization (dpf), the larvae are dependent on exogenous food. The first white adipocytes arise in the pancreatic area, underneath the swim bladder, as early as eight dpf. The formation of adipocytes appears to be correlated with the size of the larvae rather than age, suggesting body-length dependent lipid storage in adipocytes [18,21]. During early stages of development, zebrafish are optically transparent which allows in vivo monitoring of adipocyte formation and fatty acid uptake. Lipid droplets within the adipocytes can be visualized by staining developing larvae with the dye Oil Red O or with specific fluorescent dyes such as Nile Red or LipidGreen [18,22,23]. However, use of these stains has considerable drawbacks such as high photobleaching rates, strong background staining and difficulty to quantify, which can lead to misinterpretation of lipid content [24]. An imaging method that measures lipids in a label-free and quantitative manner would be highly beneficial for this research field.

Stimulated Raman Scattering (SRS) microscopy is a high-resolution chemical imaging method that is quantitative, background-free and label-free [25]. SRS has been used to image various cell cultures and model organisms such as *C. elegans*, *Xenopus laevis*, mice and zebrafish [26,27,28]. Unlike fluorescence imaging, labeling is not required because the contrast is derived from specific molecular vibrations of endogenous compounds. In SRS, two different colored laser beams are incident on a sample. If the photon energy difference between the two colors matches a molecular vibration of a molecule, energy can be transferred from one beam to the other. By applying amplitude modulation to one of the beams, the resulting intensity changes in the other beam can be measured. The efficiency of this process is a direct measure for the number of molecules of interest in the focal volume. Combined with laser scanning microscopy, this technique allows for fast and sensitive imaging with sub-micrometer resolution. In order to obtain a better understanding of adipogenesis in zebrafish larvae, high-resolution imaging like SRS microscopy would be particularly useful.

In this study, we applied SRS microscopy for the first time to determine adipocyte numbers and visualize changes in lipid volumes within zebrafish adipocytes. We also compare the SRS method to a more conventional method, i.e., lipid staining with fluorescent LipidGreen. To this end, we first established the SRS methodology by imaging baseline changes in zebrafish adipogenesis after exposure to a high fat or high carbohydrate diet from 6–15 dpf. In order to determine the effects of early life stage exposure to environmental chemicals on adipogenesis, we exposed zebrafish embryos during early embryonic development (0–6 dpf) to a non-toxic concentration of two environmental chemicals, and reared the larvae under standard diet conditions to 15 dpf. We selected the organotin pesticide tributyltin (TBT) and the chlorinated organophosphate flame retardant Tris(1,3-dichloroisopropyl)phosphate (TDCiPP) as test chemicals. Rodent studies with TBT have shown that mice exposed in utero to low concentrations show increased adiposity as adults [5,29]. In addition, zebrafish exposed during larval stages to TBT show higher levels of adiposity and lipid accumulation [30,31]. TDCiPP was selected because it is increasingly used as a flame retardant to replace polybrominated diphenyl ethers [32], suggesting high potential risk for human exposure [33]. TDCiPP is a known agonist of estrogen receptor α and increases mRNA expression of *pparα*-centered gene networks including upregulation of the *pparg* gene [34]. Preliminary data from our lab suggest that zebrafish exposed to TDCiPP during larval stages (9–15 dpf) have increased adiposity [35]. In the current study, we also examined putative molecular mechanisms underlying the changes in adipocyte number and size by measuring the mRNA expression of a panel of genes involved in adipogenesis and adipose tissue-gut-brain pathways.

## 2. Results

### 2.1. Diet Dependent Adipogenesis in Zebrafish Larvae

**Figure 1:**
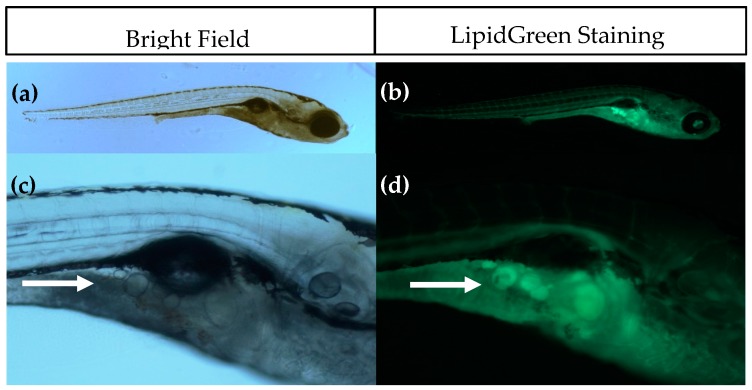
Imaging of adipocytes using Bright Field (**left**) and fluorescence microscopy after LipidGreen staining (**right**). (**a,b**) Representative images of a 15 days post fertilisation (dpf) larvae (5× magnification); (**c,d**) Close-up of the pancreatic area where the first adipocytes develop. Adipocytes are indicated with a white arrow (16× magnification).

**Figure 2:**
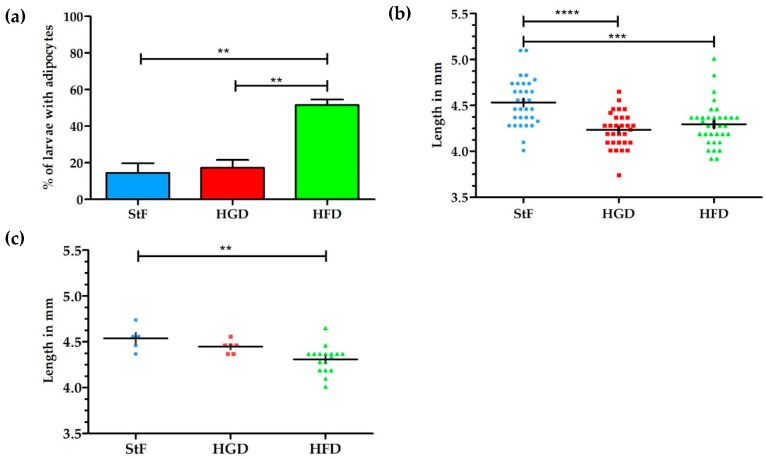
Effect of different diets on adipogenesis and growth of larvae. (**a**) Average percentage of larvae with adipocytes after feeding from 6–15 dpf with standard feed (StF), high glucose diet (HGD) or high fat diet (HFD); (**b**) Length (mm) distribution of larvae at 15 dpf. (StF: *n* = 32; HGD: *n* = 30; HFD: *n* = 33); (**c**) Length (mm) of larvae with adipocytes. (StF: *n* = 5; HGD: *n* = 6; HFD: *n* = 17) (** *p* ≤ 0.05, *** *p* ≤ 0.01, **** *p* ≤ 0.0001). Error bars represent Standard Deviation.

Larval zebrafish were fed with diets containing elevated fat or carbohydrate content, and adipogenesis was visualized at 15 dpf by both conventional fluorescent lipid staining (LipidGreen, Figure 1) and SRS microscopy. Analysis of fluorescent stained larvae at 15 dpf showed that under standard feeding (StF) conditions, 14% of larvae (average of 3 independent experiments) developed adipocytes, which is similar to the 19% of larvae that developed adipocytes after feeding with elevated carbohydrates (high glucose diet; HGD) (Figure 2a). By contrast, feeding a high fat diet (HFD) resulted in a significantly increased number of larvae with adipocytes (52%) (Figure 2a). At 15 dpf, larvae fed with HGD and HFD were significantly smaller in length compared to larvae fed with StF (Figure 2b). When considering exclusively the larvae that developed adipocytes, only larvae fed with HFD were significantly smaller in length (Figure 2c). Analysis of the length at 6, 8, 12 and 15 dpf revealed that larvae fed with high caloric diets showed reduced growth over time (Figure S1).

**Figure 3:**
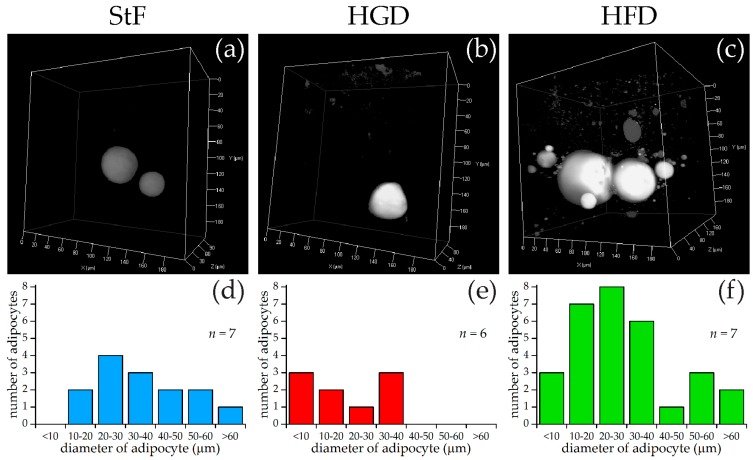
SRS imaging of adipocytes in zebrafish larvae exposed to different diets. (**a**–**c**) Representative images of volumes of SRS lipid measurements for standard diet (StF), high glucose diet (HGD) or high fat diet (HFD), respectively; (**d**–**f**) Frequency of adipocytes in different size classes following StF (*n* = 7), HGD (*n* = 6) and HFD (*n* = 7) respectively, determined by an automated image processing algorithm in MATLAB.

For SRS imaging, larvae were selected which showed adipocyte formation at 15 dpf after visual inspection under bright field microscopy. Representative images of each diet group are shown in Figure 3a–c, along with approximate diameters of the adipocytes per group (Figure 3d–f). These diameters were calculated from the volumes of the adipocytes determined by the volume analysis processing software (see Materials and Methods), assuming perfect spherical shapes. A minimal region threshold of about 5 µm in diameter was chosen for the analysis software, so very small adipocytes were not counted.

In the StF group, larvae had on average 2.0 adipocytes per fish (*n* = 7), which was comparable to larvae from the HGD group that had on average 1.5 adipocytes per fish (*n* = 6). Larvae from the HFD group had on average 5.0 adipocytes per fish (*n* = 7). The intensity of the adipocyte SRS signal in the control diet group appeared to be lower than in the HFD group (Figure 3a vs. Figure 3c). Comparison of the sizes of the adipocytes showed that adipocytes from the HGD group were notably smaller than the other two diets, which was reflected in the total adipocyte volume per fish, which is 0.1–27 pL for the HGD group compared to 9–281 pL for the StF group and 13–308 pL for the HFD group. Though StF and HGD larvae showed a similar distribution of all sizes of adipocytes, the HFD group clearly had more adipocytes in all size ranges. An overview of all size and volume measurements can be found in Table S1.

#### Gene Expression Analysis

We performed quantitative Polymerase Chain Reaction (qPCR) analysis at selected time-points during adipogenesis using a custom-developed obesity array including genetic markers for adipogenesis (adipogenic markers) and the adipose-gut-brain network. A complete list of markers is shown in Table S2.

**Figure 4:**
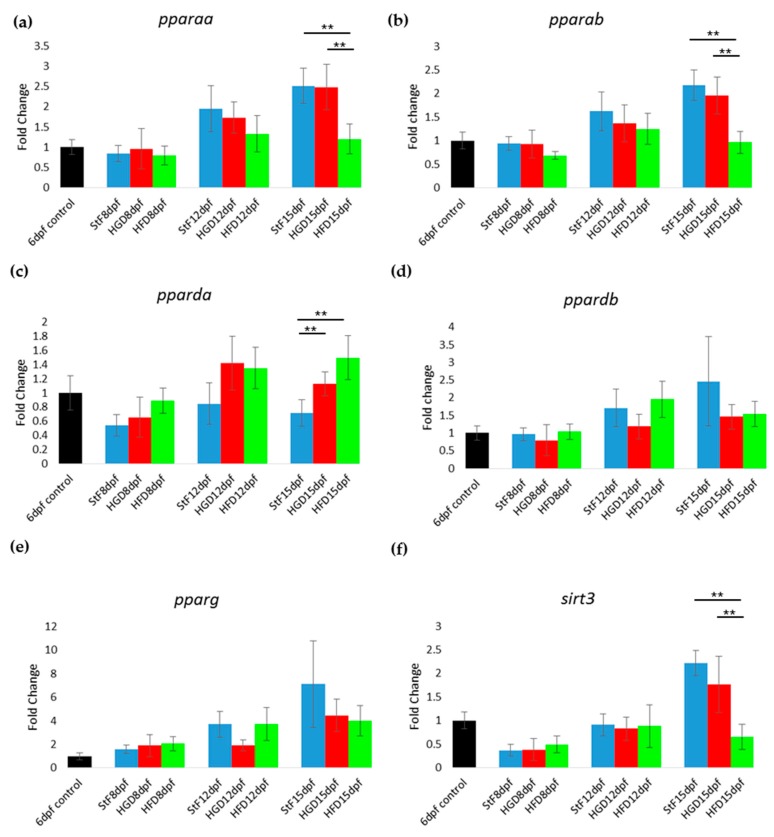
Gene expression of adipogenic markers during adipogenesis after exposure to different diets. (**a**–**f**) Fold change in expression at 8, 12 and 15 days, relative to mRNA sampled from embryos at 6 days post fertilization (6 dpf control). Larvae were fed with standard diet (StF), high glucose diet (HGD) or high fat diet (HFD) (** *p* ≤ 0.05) (*n* = 4 (each sample contained 10 larvae)). Error bars represent SEM.

In zebrafish larvae on StF or HGD, the expression of most adipogenic markers increased in an age-dependent way, from 8 to 15 dpf (Figure 4). Larvae on HFD generally showed lower expression of adipogenic markers than StF or HGD (Figure 4). For example, at 15 dpf, mRNA expression of *pparaa* and *pparab* was significantly lower in larvae fed HFD compared to StF or HGD (Figure 4a,b). Interestingly, *pparda* was the only gene in the PPAR family that was significantly elevated after HFD at 15 dpf (Figure 4c). The expression of both *ppardb* and *pparg* was not significantly changed by different diets (Figure 4d,e). Expression of *sirt3*, a member of the sirtuin family, was also significantly lower in HFD larvae (Figure 4f).

**Figure 5:**
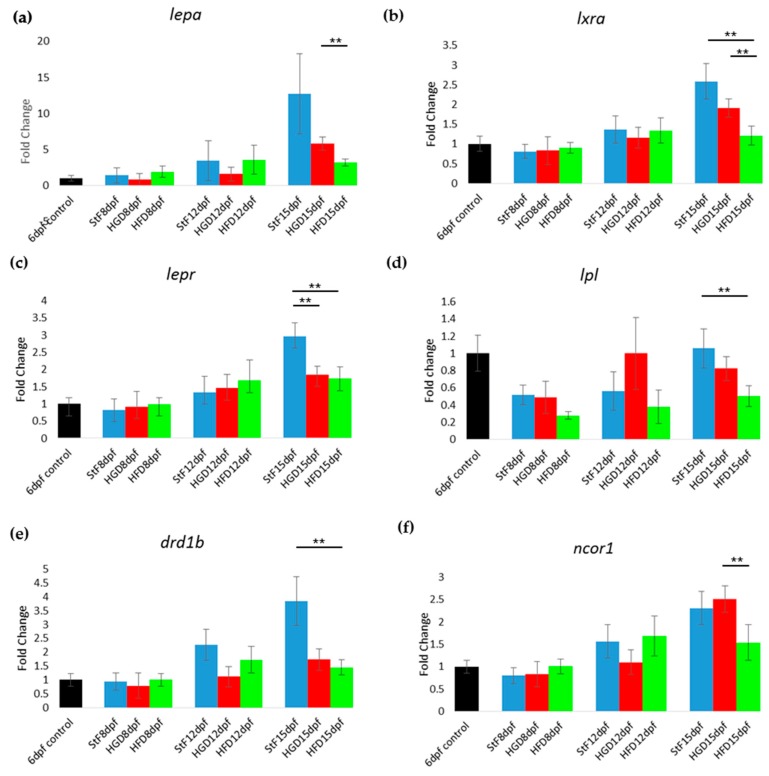
Gene expression of hormones and receptors of the adipose tissue-gut-brain pathway during adipogenesis. (**a**–**f**) Fold change in expression at 8, 12 and 15 days, relative to mRNA sampled from embryos at 6 days post fertilization prior to the start of feeding (6 dpf control). Larvae were fed with standard diet (StF), high glucose diet (HGD), or high fat diet (HFD). (** *p* ≤ 0.05). (*n* = 4 (each sample contained 10 larvae)). Error bars represent SEM.

Furthermore, the expression of genes coding for receptors and hormones of the adipose tissue–gut-brain pathway such as *lepa*, *lepr*, *lxra* (*nr1h3*), *lpl* and *drd1b* was assessed. The expression of these genes increased with developmental stage in larvae fed with StF (Figure 5a–e). This age-dependent effect was not seen in HGD and HFD larvae, where expression levels of these genes were lower compared to StF. At 15 dpf, expression of *lxra* (*nr1h3)*, *lepr*, *lpl* and *drd1b*, was significantly lower in HFD larvae relative to StF (Figure 5b–e). *lepr* was also significantly downregulated relative to StF in HGD larvae (Figure 5c). In both StF and HGD larvae, the expression of *ncor1* was increased over time, a trend that was not observed in HFD larvae (Figure 5f).

**Figure 6:**
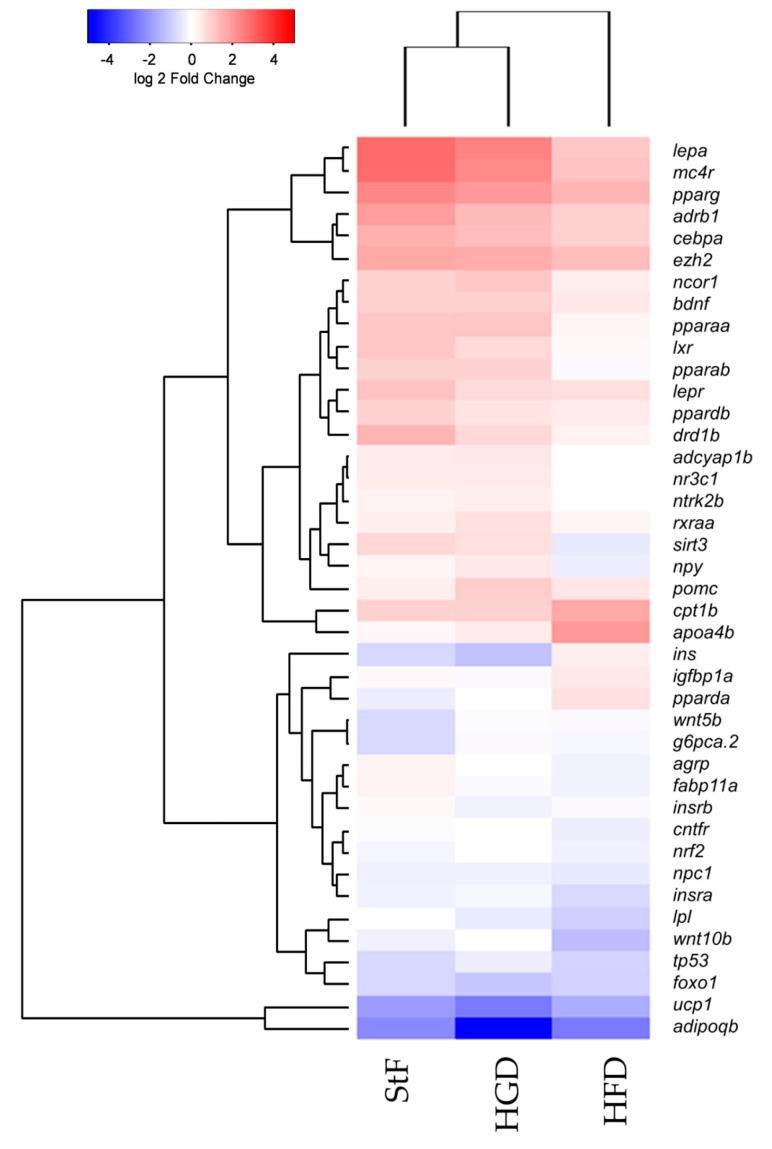
Hierarchical clustering of gene expression of genes involved in adipogenesis and adipose-gut-brain pathway. Comparison of gene expression at 15 dpf between samples of standard feed (StF), high glucose diet (HGD) and high fat diet (HFD) groups (Euclidean distance), normalized to the 6 dpf control.

In addition to the analysis of specific genes at different time points during development, we performed gene expression analysis using an obesity array in larvae at 15 dpf. We investigated the differences in gene expression between the different diet groups using an overall comparison approach which is visualized in a heatmap by hierarchical clustering of gene expression (Figure 6). In general, this gene expression analysis showed that StF and HGD are closely related and have a similar expression pattern, although HGD larvae were shorter in length, suggesting no difference in development progression. HFD larvae, which were similar in length to HGD larvae, exhibited a distinct gene expression pattern, suggesting that diet, and not differences in developmental stage, affected gene expression. This analysis showed that a number of genes involved in adipogenesis pathway and Ppar signaling were activated in larvae from the StF and HGD diet groups, while in larvae fed with HFD showed reduced expression (Figure 6).

### 2.2. Analysis of Adipogenesis after Early Life Exposure to Environmental Chemicals

**Figure 7:**
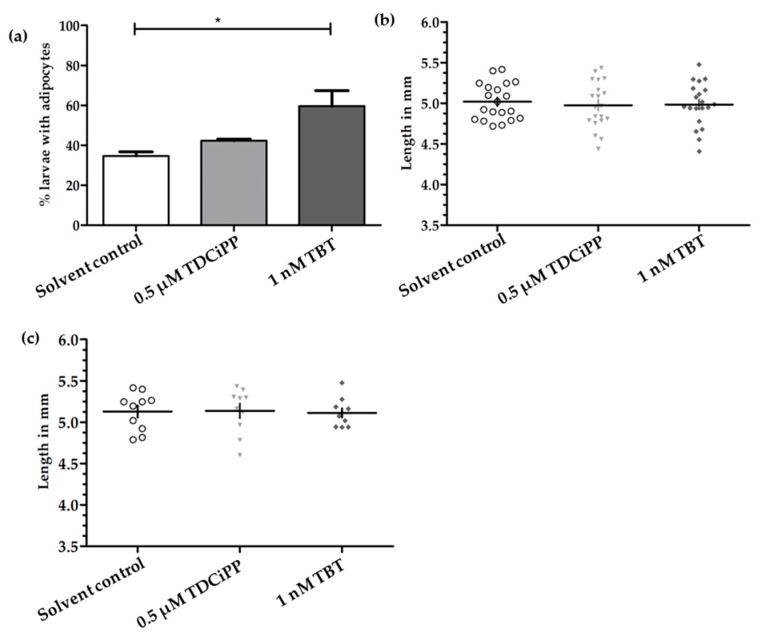
Effect of developmental exposure to environmental chemicals on adipogenesis and growth of larvae. (**a**) Presence of adipocytes in 15 dpf larvae after developmental (0–6 dpf) exposure to solvent control (0.01% DMSO), 0.5 μM TDCiPP, or 1 nM TBT; (**b**) Length (mm) distribution of larvae at 15 dpf (*n* = 20); (**c**) Length (mm) of larvae with adipocytes (*n* = 10) Error bars represent Standard Deviation. (* *p* ≤ 0.05).

In a preliminary study, zebrafish embryos were exposed to a concentration range of the putative environmental obesogens TDCiPP and TBT from 0–6 dpf in order to identify a concentration that caused no phenotypical effect on development (Figure S2). We then selected one non-toxic concentration of TDCiPP (0.5 μM) and TBT (1 nM); embryos were exposed from 0–6 dpf to this concentration, and reared under a StF regime from 6–15 dpf. In the solvent control (0.01% DMSO) group, on average 35% of the larvae (*n* = 2 experiments) developed adipocytes at 15 dpf (Figure 7a). A similar percentage was observed after exposure to 0.5 µM TDCiPP (42%). After exposure to 1 nM TBT, the percentage of larvae that developed adipocytes was significantly increased relative to the solvent control (60%). We also measured the length of larvae at 15 dpf in the different groups but found no difference between treatments when considering all larvae (Figure 7b) or exclusively larvae with adipocytes (Figure 7c).

**Figure 8:**
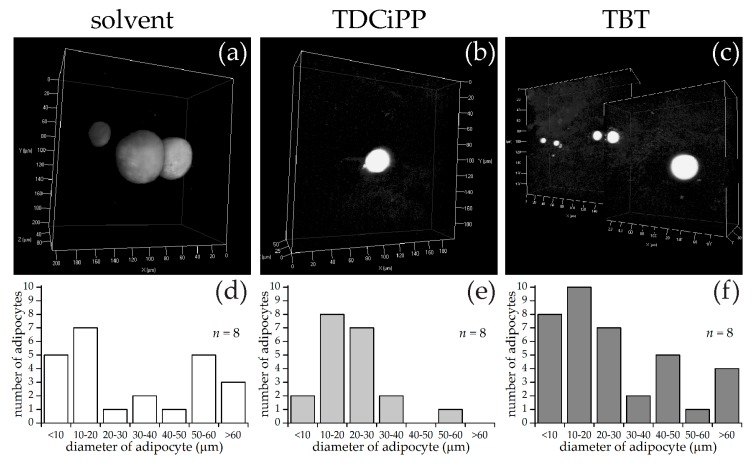
SRS imaging of adipocytes in fish exposed to environmental chemicals. (**a**–**c**) Representative images of volumes of SRS lipid measurements following solvent control (0.01% DMSO), 0.5 µM TDCiPP, or 1 nM TBT exposure, respectively; (**d**–**f**) Frequency of adipocytes in different size classes of solvent control (*n* = 8), TDCiPP (*n* = 8) or TBT (*n* = 8) respectively, determined by an automated image processing algorithm in MATLAB.

Using the same approach as described above for diets, larvae at 15 dpf were selected for SRS imaging. Representative images of each exposure group are shown in Figure 8, along with measurements of the diameter of the adipocytes per fish of adipocytes larger than 5 µm in diameter. In the solvent control group, the fish had on average 3.0 adipocytes per fish (*n* = 8), in the TDCiPP group 2.5 adipocytes per fish (*n* = 8) and in the TBT group 4.6 adipocytes per fish (*n* = 8). The histograms of adipocyte sizes show no clear differences between the three groups. The total adipocyte volumes range from 4–1235 pL for the control group, 1–91 pL for the TDCiPP group and 8–550 pL for the TBT group. The range for the control group was skewed by one fish with an unusually large 122 µm diameter adipocyte. An overview of all size and volume measurements can be found in Table S3.

#### Gene Expression Analysis

**Figure 9:**
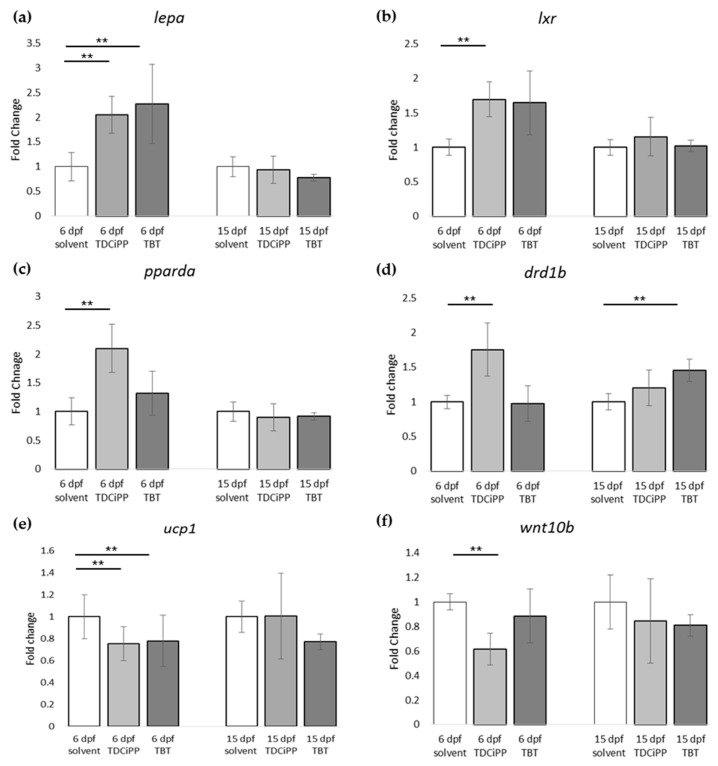

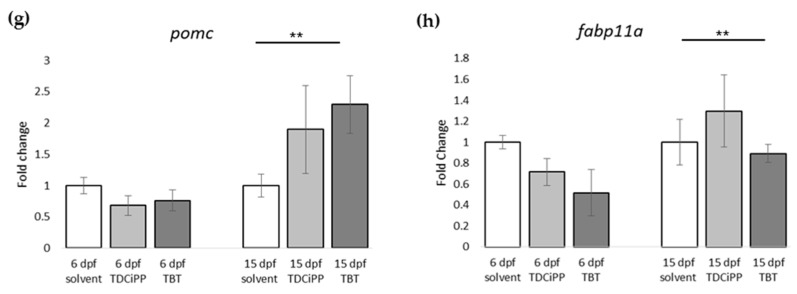
Gene expression of hormones and receptors after early exposure to environmental chemicals. (**a**–**h**) Fold change in expression of genes (normalized to solvent control at either 6 dpf or 15 dpf) were measured with qPCR using the obesity array. Samples were taken from two independent exposure experiments (*n* = 3 per experiment); (** *p* ≤ 0.05). Error bars represent SEM.

For gene expression analysis, larvae were analyzed either directly after exposure (6 dpf), or after maintaining the larvae on StF from 6 to 15 dpf. We analyzed the same genes using the obesity array as described earlier. Direct exposure to TDCiPP or TBT resulted in significantly increased expression the *lepa* gene relative to the solvent control at 6 dpf (Figure 9a). TDCiPP exposure resulted in significantly higher expression of *lxr*, *pparda and drdb1* at 6 dpf (Figure 9b–d). *fabp11a* was the only gene that showed significantly lower expression after exposure to both TBT and TDCiPP (Figure 9e). The expression of *wnt10b* was significantly reduced in larvae exposed to TDCiPP at 6 dpf (Figure 9f). Gene expression changes of most of these genes was no longer changed when measured at 15 dpf. In general, most genes at 15 dpf were expressed at a level comparable to larvae from the control group, with the exception of *drdb1* and *pomc*, which were significantly higher expressed in larvae exposed to TBT, and *ucp1*, which was significantly reduced after developmental TBT exposure (Figure 9d,g,h).

**Figure 10:**
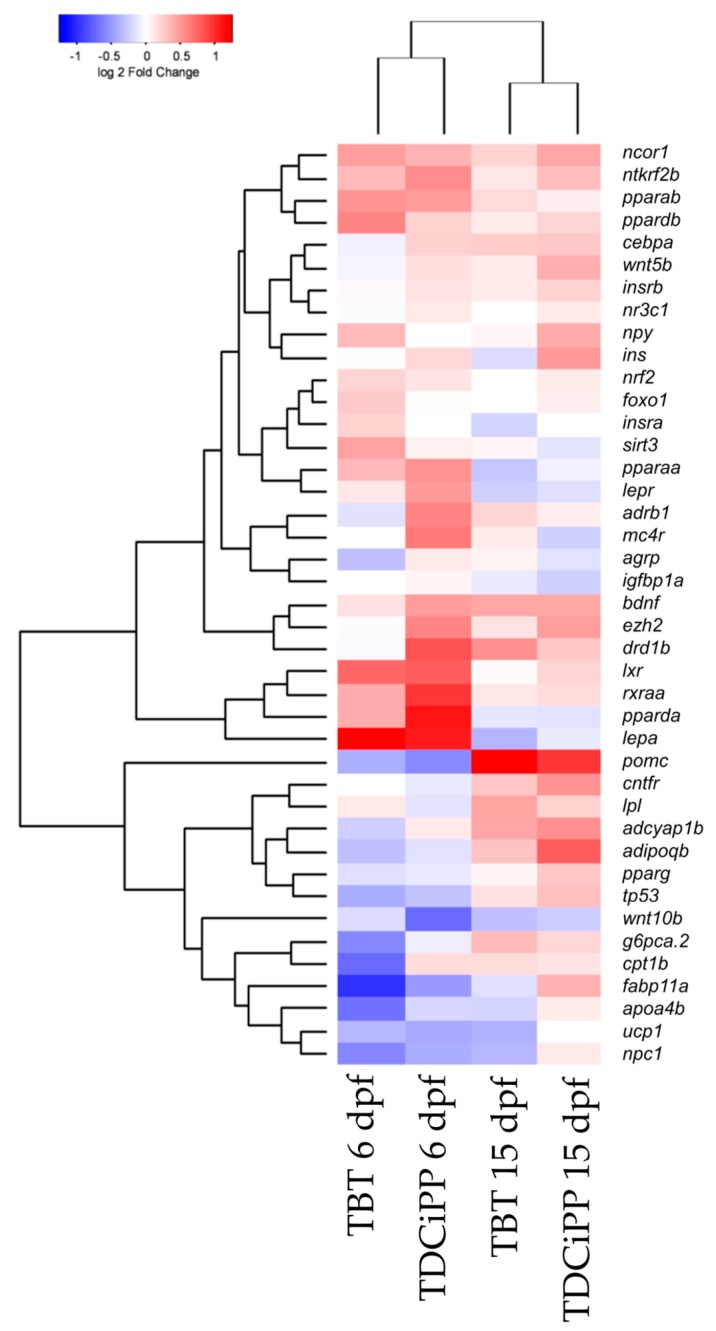
Hierarchical clustering of gene expression after exposure to environmental chemicals. Comparison of gene expression at 6 and 15 dpf after exposure from 0–6 dpf to 1 nM TBT or 0.5 μM TDCiPP. The gene expression was normalized to the solvent control per time point (Euclidean distance).

Hierarchical clustering of the gene expression data sets shows that both compounds cluster together at 6 and 15 dpf, which indicates that the compounds exhibit a similar gene expression pattern at these time points (Figure 10). More pronounced changes in gene expression were found at 6 dpf compared to 15 dpf, indicating that direct exposure to the chemicals altered gene expression (Figure 10).

## 3. Discussion

The aim of this study was to examine the effects of different diets and chemical exposures on adipogenesis in zebrafish. SRS was applied to image adipocyte development in zebrafish for the first time. SRS allows direct visualization without the need for labeling, and provides additional insight into adipocyte number, size and volume. In this study, zebrafish larvae fed a high fat diet (HFD) from 6 to 15 dpf showed a threefold increase in the prevalence of larvae with adipocytes in comparison to standard feed (StF) or a diet with high glucose (HGD). Although an increased prevalence of adipocytes in zebrafish larvae on HFD has been described earlier [36], SRS imaging showed that larvae fed with HFD had on average more adipocytes of all size distributions in comparison to larvae in the StF and HGD groups, suggesting both adipocyte hyperplasia and hypertrophy after HFD. SRS imaging also suggested a possible increase in the intensity of the SRS signal in the HFD group compared to the control group, indicating a higher density of lipids in these adipocytes. Future exploitation of SRS technology could allow to us to determine the nature of the differences in lipid density.

Interestingly, high caloric diets showed an impact on growth, as both larvae from HFD and HGD groups had a lower growth rate and were significantly smaller in length at 15 dpf than the control group. Furthermore, larvae from the HFD group developed adipocytes at a smaller standard length. The smaller length of the larvae on HFD is presumably caused by a shift in energy balance towards processing calories in order to store excess energy in adipocytes, thereby diverting energy from growth. Previous studies have indicated that the number and development of adipocytes is correlated with size of the larvae rather than age, suggesting that a minimum body length is needed to trigger adipogenesis [18,21]. Our results contradict these findings, suggesting that diet may be more important than body length in determining when adipocytes first start to differentiate.

While it is has been suggested that zebrafish share common pathways with mammals in terms of lipid metabolism and lipid storage in adipocytes [37], relatively little is known about the function and expression of key regulators during zebrafish adipogenesis. In this study, gene expression analysis focusing on important regulators in the transcriptional network of adipogenesis was performed in order to provide more insight into the molecular mechanisms of diet-induced adipogenesis in zebrafish. In humans and rodents, the three PPARs (PPARA, PPARD and PPARG) are involved in lipid metabolism, fatty acid oxidation and glucose homeostasis. PPARG is also known as the master regulator in adipocyte differentiation. In zebrafish, five orthologs of PPARs have been identified (reviewed in Den Broeder et al., 2015 [19]); however, their exact functions have not been elucidated. In our study, the expression of *pparg* at 8, 12 and 15 dpf was not significantly changed upon feeding with a high caloric diet, despite the increased adipocyte number and size in HFD larvae. This is surprising, as our HFD consisted of diluted egg yolk containing saturated and unsaturated fats, which are endogenous ligands of mammalian PPARs. A possible explanation could be that our egg yolk solution contained a high ratio of omega3:omega6 polyunsaturated fatty acids, which has been shown to decrease PPARG mRNA level in mice fed with HFD [38,39]. Another possibility may involve the reported differences in the amino acid sequence of the ligand binding domain of zebrafish Pparg, which may result in a different receptor affinity for fatty acids and fatty acid derivatives compared to human and rodent PPARG [40]. We can also not exclude that Pparg may not be the master regulator of adipogenesis in zebrafish, as other family members can have a redundant function, a common feature of cyprinid fish [41]. Accordingly, the expression of *pparda* was significantly increased in HFD larvae, suggesting that this gene may play a role in adipogenesis in zebrafish. Clearly more research is needed to investigate the expression *ppar* genes and proteins in developing zebrafish larvae and determine their role in adipogenesis.

Other genes in the adipogenic pathway showed repression after HFD in zebrafish, such as the liver X receptor *lxra* (*nr1h3*) and the leptin receptor gene *lepr*. In mammals, NR1H3 (LXRa) stimulates adipogenesis by increasing the levels of PPARG [42], and LEPR is expressed in the hypothalamus and binds leptin, the hormone excreted by adipose cells that in mammals plays an important role in appetite regulation [43]. In mice, mutation of both leptin and the leptin receptor is associated with obesity and disturbed glucose homeostasis [44,45]. *lepr* may also play an important role in glucose homeostasis in the zebrafish, as *lepr* mutant fish show increased insulin gene expression and higher β-cells numbers [46]. Larvae in the HFD group showed reduced expression of the nuclear receptor corepressor 1 (*ncor1*), which is one of the co-factors for the PPAR protein family members. In rodents, high fatty acid levels decrease the expression of NCoR1, while insulin and high-glucose levels increase its expression [47]. Also the expression of the dopamine receptor 1 b (*drd1b*) was lower in larvae fed with HFD. DRD1B is one member of the dopamine receptors (D1–D5) in the hypothalamus, a class of receptors that are involved in the regulation of food intake [48].

Feeding larvae a high glucose diet did not affect the number of adipocytes compared to standard diet, though SRS imaging revealed that adipocytes from the HGD group were smaller in size. There were also few differences in gene expression between HGD and standard feed. It is possible that the concentration of glucose, or the duration of the feeding, was not sufficient to induce significant gene expression changes, though embryo exposure with similar concentrations of glucose have shown changes in the insulin pathway [49]. Further studies with multiple glucose concentrations and gene expression analysis of insulin pathways are recommended.

In addition to the effects of diet on adipogenesis, we studied the effects of early life exposure to the environmental chemicals TBT and TDCiPP at low, non-toxic concentrations that did not cause effects on growth or development. We show for the first time that exposure to a low nanomolar concentration of TBT during early development stimulates adipogenesis in zebrafish, resulting in a doubling of the number of larvae with adipocytes later in life. SRS imaging showed fish exposed to TBT had on average more adipocytes per fish than in the solvent control fish, but did not show a different size distribution of these adipocytes, suggesting hyperplastic effects of TBT. The obesogenic effect of developmental exposure to TBT has been shown before in rodents [5]. In zebrafish, exposure from 3–11 dpf with low concentrations of TBT (0.1, 1 nM) resulted in an increased lipid accumulation [30]. Also exposure of larvae, which had already developed adipocytes, to 50 nM TBT for 24 h resulted in increased adiposity [31]. In mammals and *Xenopus*, the effect of TBT has been linked to its binding to Pparg/RXR, thereby promoting adipocyte differentiation [5]. However, we did not measure a significant change in expression of *pparg* or *rxraa.* Our gene expression data show an upregulation of *lxr* directly after exposure to TBT at 6 dpf. Interestingly, we measured increased expression of *pomc* mRNA at 15 dpf in the TBT group, a hypothalamic factor involved in feeding behavior. We did not measure food uptake in this study, and cannot exclude that TBT changed feeding behavior; it would be useful to measure food uptake in future studies. Our results are important because the concentration we tested was extremely low, and while TBT is no longer widely used as an antifoulant, its presence in the environment is still detected [50].

Exposure to 0.5 µM TDCiPP early in development did not result in effects on adipogenesis. The prevalence of adipocytes was not significantly increased, and SRS imaging did not show an increase in the number of adipocytes per fish or a different size distribution. However, preliminary studies in our laboratory suggest that exposure to this chemical at later stages (9–15 dpf) results in increased lipid accumulation in visceral tissue (Kopp et al., manuscript in prep). Changes in gene expression of *lepa*, *pparda* and *drd1b* were found in 6 dpf embryos directly after TDCiPP exposure, indicating an effect of early exposure on a genetic level. Our hierarchical cluster analysis suggests that most genes show a similar gene expression pattern compared to TBT. Future studies should include a more detailed dose-response study with this and other putative obesogens in which the longer-term effects on adipogenesis are examined.

We observed that the solvent control group had a higher prevalence of larvae with adipocyte development than the control group (StF) in the diet experiments, even though we used the same diet in both experiments. It is unlikely that such a low concentration of DMSO (0.01%) had an effect on adipocyte development [51,52]. The diet and chemicals exposure experiments were performed separately and different batches of zebrafish eggs from different parents were used, indicating that differences in adipocyte prevalence are likely caused by inter-experimental biological variation and therefore we do not directly compare the results of the diet experiments with those of the environmental chemicals experiments.

The introduction of SRS imaging to adipocyte research in zebrafish is one of the strengths of this study. Fluorescence imaging with lipid stains such as LipidGreen can be difficult to interpret and does not give quantitative information (see Figure 1). Compared to fluorescence imaging methods, SRS has several advantages, including lack of photobleaching, inherent 3D sectioning possibilities and low levels of photodamage due to near-IR wavelengths. Some of these advantages are shared with two-photon excited fluorescence and confocal fluorescence, but fluorescent labeling would still be required with these techniques. Staining with fluorescent dyes is not necessary in SRS, because the contrast is derived from intrinsic vibrational energy levels. This not only eliminates the time-consuming staining process, but also prevents incorrect interpretations due to incomplete staining or unexpected behavior of the stain. The additional benefit of SRS imaging combined with semi-automatic adipocyte counting and volume computation has been demonstrated in this work. Compared with conventional Raman microscopy, the imaging rate of SRS can be orders of magnitude higher, but for only one vibrational band at the time [53,54]. SRS in principle is a quantitative method, but quantification of adipocyte content was not possible in these experiments due to the fixation with paraformaldehyde which adds to the SRS signal. Fixation of the larvae was necessary to perform bleaching to reduce pigmentation, as it is not feasible to perform SRS imaging through this highly scattering layer. Attempts to use the Caspar strain zebrafish were unsuccessful as they showed a delayed adipocyte development, indicating an altered metabolism due to lack of pigment cell formation.

In conclusion, this study revealed that adipogenesis in zebrafish is increased early in life by developmental exposure to the environmental obesogen TBT. High fat diet early in life also significantly increased adipogenesis. Studies of the effects of different diets on adipogenesis revealed how powerful SRS imaging is to determine changes in adipocyte size, volume and numbers in real time. Although the zebrafish is a promising model for studying vertebrate adipogenesis, gene expression analysis revealed that the expression of adipogenic genes such as *pparg* in response to diets or obesogenic chemicals may differ from higher vertebrates. More research is needed to better understand the relevant genes and proteins that regulate adipogenesis in zebrafish. To this end, research with adipocyte-specific transgenic fish lines or knock-outs of homologous genes known to be important in human adipocyte development is recommended. Ultimately, the results of our study emphasize the importance of identifying chemical effects on adipogenesis, as reducing developmental exposures to obesogenic chemicals is an important preventative strategy in addressing the global obesity epidemic.

## 4. Materials and Methods

### 4.1. Zebrafish Maintenance

Wildtype zebrafish (AB strain) were maintained in tanks in a continuous flow system (Aqua Schwarz GmbH, Göttingen, Germany) with daily 15% water exchange, at 26 °C under a light/dark cycle of 14 h light/10 h dark. Adult fish were fed three times per day, two times with Tetramin flakes and one time with cultured *Artemia salinas*. The day before mating, female and male fish (1:1 ratio) were placed in spawning boxes and separated by a net. The next morning, female and male were put together to mate and embryos were collected and transferred to petri dishes (60 larvae per petri dish) containing E3 medium (5 mM NaCl, 0.17 mM KCl, 0.33 mM CaCl_2_·2H_2_O, 0.33 mM MgSO_4_·7H_2_O) and incubated at 26 °C.

The studies described in this manuscript were approved by the Committee on the Ethics of Animal Experiments of the VU University Amsterdam (DEC code: IVM 11-01 (Approval date: April 2011); IVM 14-01 (Approval date: November 2014)) in accordance with the law of the Dutch Parliament.

### 4.2. Feeding Protocol

**Figure 11:**
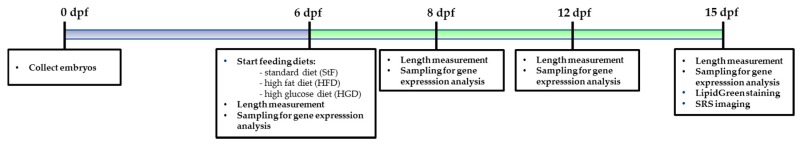
Schematic representation of the procedure used to analyze the effects of different diets on adipocyte development in zebrafish larvae.

Larvae for the feeding experiment were preselected at 5 dpf for the presence of an inflated swim bladder, a measure of health and vitality. At 6 dpf, larvae were randomly transferred to mini tanks in 100 mL E3 medium with maximum 40 larvae per tank. Larvae were assigned into different groups and fed with either standard feed (StF), high fat diet (HFD) or high glucose diet (HGD). We performed three independent feeding experiments, and each experiment contained in three biological replicates per diet group. Tanks were refreshed every other day. The timeline of the diet experiments is illustrated in Figure 11.

The standard diet group was fed twice a day with 2.0 mL *Tetrahymena pyroformis* (TH) supplemented with dry food (Sera micron). TH is a small unicellular organism found in fresh water ponds, which was cultured in sterile Protease Peptone Yeast Extract Medium (PPY) containing 20.0 g Protease peptone; 2.5 g Yeast extract in 1.0 L distilled water. To start a culture, 6 mL TH stock culture was inoculated into 200 mL PPY culture. The culture medium was kept for 3–4 weeks at 16 °C. TH was harvested by filtering TH culture over a Whatmann paper filter. Filter paper was rinsed with Prescotts & James’s Solution (PJ), and TH remaining on the filter was taken up in 100 mL PJ medium. TH solution was kept in the refrigerator at 4 °C for maximum of 7 days. The fat percentage of TH was <0.05%.

The HFD group was fed with 2.0 mL boiled egg yolk solution twice a day, together with dry food. Chicken eggs purchased at a local grocery store were boiled for 5 min, and the egg yolk was taken out. HFD solution was made by adding 1 g of egg yolk to 15 mL E3 medium. Egg yolk was mashed and the solution was shaken vigorously to ensure suspension. Larger particles were allowed to settle to the bottom of the tube and the upper, less dense portion was used to feed the developing larvae. The fat percentage of egg yolk solution, after further dilution to 100 mL of E3 medium, was 0.4%.

Larvae receiving HGD were reared in a final concentration of 20 mM glucose solution (Sigma-Aldrich, CAS 50-99-7, G7021, St. Louis, MO, USA), which was prepared in 100 mL E3 medium. Feeding was supplemented with dry food.

### 4.3. Early Life Exposure to Environmental Chemicals

**Figure 12:**
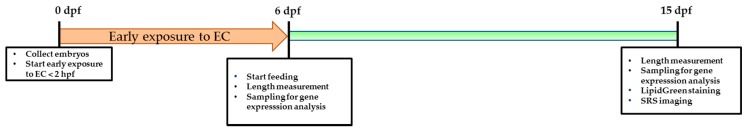
Schematic representation of the procedure used to analyze the effects of environmental chemicals (EC) on adipocyte development in zebrafish larvae.

TDCiPP (13674-87-8, purity > 96%) was kindly provided by Prof. A. Bergman, SweTox. TBT was purchased at Sigma-Aldrich (CAS: 1461-22-9, purity 96%). The chemicals were dissolved to a 100 mM stock solution in dimethylsulfoxide (DMSO, Across, CAS: 67-68-5). The environmental chemicals were dosed in E3 medium at a solvent volume of 0.01% DMSO. The concentrations of environmental chemicals selected were based on previous embryo toxicity tests and showed no effects on embryo development and survival from 0 to 6 dpf (see Figure S2). Embryos <2 hpf were randomly selected (*n* = 40) and exposed in three biological replicates to a selected non-toxic concentration of a single compound (TDCiPP 0.5 μM; TBT 1 nM) in a total amount of 10 mL in a six well plate. We performed two independent chemical exposure experiments. At 6 dpf, larvae were transferred to a mini tank containing 100 mL E3 medium free of chemical compounds and larvae were fed with standard feed. Medium in the tanks was refreshed daily. The timeline of the chemical exposure experiments is illustrated in Figure 12.

### 4.4. Length Measurement

A subsample of larvae from each diet or exposure group was randomly selected, anaesthetized with MS222 (Tricaine, CAS: 888-86-2, Sigma-Aldrich, St. Louis, MO, USA and transferred to 3% methylcellulose. Images of larvae were taken at 0.8× magnification using a Leica MZ FLIII microscope (Leica Microsystems BV, Rijswijk, The Netherlands) with a mounted camera and LAS software (LAS Software V3.6, Leica Microsystems BV, Rijswijk, The Netherlands). A picture was taken of a calibration slide with 10 mm long bar with the same magnification. Image J software was used the measure the standard length of larvae from mouth to the caudal peduncle.

### 4.5. LipidGreen Staining and Imaging of Adipocytes

LipidGreen staining was performed at 15 dpf to check for the presence of adipocytes. Larvae were fasted for 12 h before staining in order to empty the digestive tract, as the presence of food in the gut may give a high background signal, thereby making it difficult to visualize adipocytes. The protocol for LipidGreen staining was performed as described in Chun et al., 2013 [55]. LipidGreen solution with a final concentration of 1 µM was prepared in E3 medium. After staining, larvae were 3× 10 min washed with E3 medium. During staining and washes, larvae were kept protected from light. Larvae were anaesthetized with 0.15% MS222 and mounted on 3% methyl cellulose containing 0.15% MS222. Images of larvae were captured using an inverted fluorescence microscope (Leica DMIL LED with a DFC295 camera, Leica Microsystems BV, Rijswijk, The Netherlands) and Leica LAS software.

### 4.6. RNA Extraction and cDNA Synthesis

Tissue homogenization was achieved using the Precellys 24 tissue homogenizer: 2 × 30 s at 6000 rpm, and RNA extraction was performed using the Nucleo Spin 8 RNA kit (Machery-Nagel GmbH & Co. KG, Düren, Germany, Cat. No. 740465.4) with vacuum extraction. For the diet samples we used four biological replicates, using 10 pooled larvae per time point. Samples after early exposure to environmental chemicals were taken from two independent exposure experiments. From each experiment, 3 biological replicates containing 10 pooled larvae were sampled. After RNA extraction, total RNA concentration was measured using Nanodrop (ND-1000, NanoDrop Technologies, Inc., Wilmington, DE, USA) spectrophotometer. One µg of RNA was used for first-strand cDNA synthesis using the high-capapity cDNA synthesis kit (Thermo Fischer, Applied Biosystems^TM^, Foster City, CA, USA, Cat. No.: 4368814), and contained 8 mM dNTPs, random hexamers, 5 U/µL reverse transcriptase and 1.0 µg total RNA. Reaction conditions were: 10 min 25 °C, 120 min 37 °C and 5 min 85 °C.

### 4.7. Obesity Array and qPCR

In the 48-well obesity array, one blank, one interplate calibration well (known cDNA sample and a selected primer set), and four reference genes (*ef1α*, *gapdh*, *hprt1*, *nono*) were added in order to normalize our datasets. We have selected genes that are known to play a role in adipogenesis, and genes known to have a role in the adipose tissue-gut-brain pathway (see Table S2 for full list of genes and primer sequences). Primers were developed using the primer blast tool from NCBI (https://www.ncbi.nlm.nih.gov/tools/primer-blast/), with an amplicon length between 70 and 200 bp, and annealing temperatures of 60 degrees, overspanning an intron, preferably one primer overlapping an exon-exon junction. For every primer set the efficiency was determined using serial dilutions of cDNA. Primer dimer formation and/or secondary products were checked by analyzing the dissociation curves and gel electrophoresis.

qPCR reactions were performed using SYBR Green universal supermix (BioRad, 172-5121) and contained 15 times diluted cDNA and 250 nM of each primer in a final reaction of 10 µL. 48 wells of a 96 wells plate were used for each cDNA sample, allowing 2 samples per PCR plate. qPCR reaction conditions were: 15 min 95 °C followed by 40 cycles of 15 s 95 °C, 45 s 60 °C and plate analysis. The Cq determination was done by regression, and the fold change in expression (Δ∆*C*_q_) was calculated using the BioRad CFX analysis software. Significance analysis was performed on the log2 transformed values that were obtained via ANOVA analysis. Hierarchical clustering of log2 normalized fold changes was performed in R (v3.3.2) using heatmap.2 in the gplots package (v.3.0.1). Clustering was performed with Euclidean distance and complete agglomeration.

### 4.8. SRS Imaging of Adipocytes

All larvae that were selected for the presence of adipocytes were fixed in 4% paraformaldehyde (PFA) overnight. After removal of PFA, larvae were bleached in 3% hydrogen peroxide in 15 μM KOH. Larvae were transferred to 1% methylcellose for 30 min to minimize the amount of air bubbles. Larvae (*n* = 7–8) were randomly selected for SRS-imaging, and were mounted on a microscope slide using low melting point agarose in order to prevent movement during imaging.

**Figure 13:**
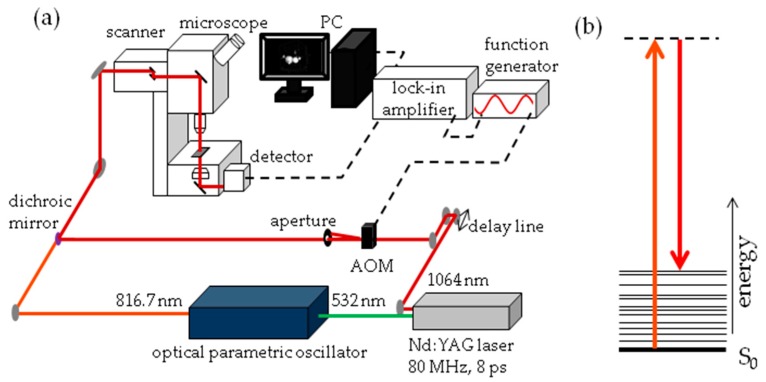
Stimulated Raman Scattering (SRS) imaging details (**a**) Schematic representation of SRS imaging set-up. AOM = acousto-optic modulator; (**b**) Jablonski energy diagram representation of SRS. The S_0_ electronic ground state interacts with the vibrationally excited state through a virtual state. The difference in photon energy between the two lasers corresponds with the 2845 cm^−1^ stretch vibration of CH_2_ groups.

The experimental set-up for SRS imaging is mainly as described previously [56] and is shown in Figure 13. In brief, laser light at 1064 nm (80 MHz repetition rate, 8 ps pulse length) is intensity modulated at 3.636 MHz with an acousto-optic modulator and overlapped with 816.7 nm light for imaging at 2845 cm^−1^. A laser power ratio of 2:1 of the modulated versus unmodulated beams is selected for all imaging, total laser power on the sample was 15 mW. A Zeiss laser scanning microscope (LSM7MP) with 32× objective (C-Achroplan W, NA = 0.85) is used to image samples with non-descanned detection in forward scattering mode. The signal is amplified with a homebuilt transimpedance amplifier before demodulation with a time constant of 100 μs in a lock-in amplifier (Stanford Research Systems SR844). The X-phase output at a sensitivity of 1 mV was supplied to the ZEN microscopy software. Recorded volumes are 200 × 200 μm (512 × 512 pixels) in *x* and *y* with a pixel dwell time of 100 μs and 25 to 66 slices in the *z* dimension, with a *z*-spacing of 3 μm. When all adipocytes of the fish did not fit into one volume, the fish was recorded in two parts, each with these dimensions. Measurement time for an entire volume was 30 to 70 min.

A custom MATLAB code was developed to compute the adipocyte volumes. Image stacks are extracted from the ZEN software and the raw data are denoised via a block-matching collaborative filtering (BM3D) method [57]. The performance of this technique is strongly dependent on the regularization parameter used in order to assure the convergence of the algorithm defined by the standard deviation of the analyzed image. In our case, the BM3D regularization parameter was computed as the average standard deviation (SD) of each 3D data set. By denoising the raw data via BM3D, the edges of the adipocyte are better preserved and blurring effects are reduced. Subsequently, the resulting denoised data are converted from intensity images into binary images using a global threshold determined with Otsu’s method [58]. The threshold used for the entire volume was determined as the average of the highest 90% of the global threshold values. This ensures that frames within the *z*-stack that do not contain any adipocytes do not contribute too strongly to the average threshold. For SRS imaging, we defined adipocytes as the regions with lipid signal larger than about 5 µm in diameter and corresponding to an adipocyte by visual inspection of location and shape. From each frame, areas smaller than 100 pixels (corresponding to areas smaller than about 5 μm diameter) are removed using a morphological operation. This 100-pixel value was determined as the optimal value to discard as many small regions of background signal as possible, without losing small adipocytes. As can be seen in Figure 3, adipocytes can be very close to each other or even touching. Therefore, a 3D watershed algorithm [59] was used to detect the crest lines of the binary volume, find the individual adipocytes and separate connected components. The output is examined and, where necessary, a 2D watershed approach is applied to individual *z*-stack slices of specific components. Oversegmented components are manually combined and components that do not represent an adipocyte are discarded. Volumes are computed from the number of pixels in the section and the voxel volume of 0.39 × 0.39 × 3 μm. The MATLAB analysis code package can be found in the Supplementary Materials.

### 4.9. Statistical Analysis

Significant differences were calculated by applying analysis of variance (ANOVA) followed by a post hoc test using GraphPad Prism 5.0 software (GraphPad Software, Inc., La Jolla, CA, USA).

## Supplementary Materials

Supplementary materials can be found at www.mdpi.com/1422-0067/18/4/894/s1.

## Abbreviations

BAT	Brown adipose tissue
dpf	Days post fertilisation
HFD	High fat diet
HGD	High glucose diet
hpf	Hours post fertilisation
qPCR	Quantitative Polymerase Chain Reaction
SL	Standard length
SRS	Stimulated Raman Scattering
StF	Standard Feed
TBT	Tributyltin
TDCiPP	Tris(1,3-dichloroisopropyl) phosphate
WAT	White adipose tissue
