# Supplementary material for: Altered Adipogenesis in Zebrafish Larvae Following High Fat Diet and Chemical Exposure Is Visualised by Stimulated Raman Scattering Microscopy
Source: Int J Mol Sci. 2017 Apr 24;18(4):894. doi: 10.3390/ijms18040894 (PMC5412473; doi:10.3390/ijms18040894)
Supplement: Supplementary file 1 [file ijms-18-00894-s001.zip › ijms-185594-supplementary materials.pdf]

Altered Adipogenesis in Zebrafish Larvae Following High Fat Diet and Chemical Exposure Is Visualised by Stimulated Raman Scattering Microscopy

Marjo J. den Broeder, Miriam J. B. Moester, Jorke H. Kamstra, Peter H. Cenijn, Valentina Davidoiu, Leonie M. Kamminga, Freek Ariese, Johannes F. de Boer and Juliette Legler

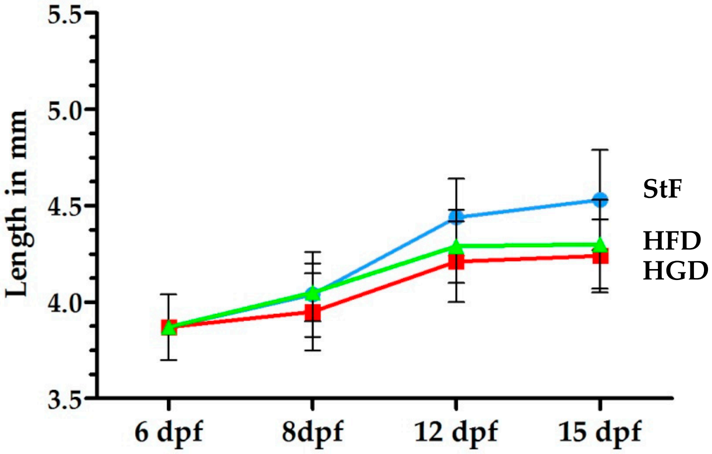

Figure S1. Growth curve from larvae fed with different caloric diets. Standard Length (mm) measurement at selected time points during zebrafish larval development. Larvae were fed with standard diet (StF), high glucose diet (HGD) or high fat diet (HFD). Error bars represent Standard Deviation.

Table S1. Overview of all adipocyte measurements of fish exposed to different diets. All images made with SRS imaging can be found in the supplementary materials.

	average number of adipocytes	average volume per fish in pL
StF	2.0	76
HGD	1.5	14
HFD	5.0	132

FISH ID	number of adipocytes	adipocyte volume in pL	approximate diameter in µm	total volume per fish in pL
StF 1	3	29	38	281
		77	53	
		174	69	
StF 2	2	5	21	45
		40	42	
StF 3	3	1	13	34
		25	36	
		8	25	
StF 4	2	28	38	39
		11	28	
StF 5	2	37	41	39
		2	17	
StF 6	1	82	54	82
StF 7	1	9	26	9

HGD 1	1	26	37	26
HGD 2	1	27	37	27
HGD 3	1	0.1	5	0.1
HGD 4	2	8	25	11
		2	16	
HGD 5	1	0.1	6	0.1
HGD 6	3	0.3	8	20
		2	14	
		19	33	
HFD 1	5	0.3	9	98
		1	13	
		58	48	
		30	39	
		9	26	
HFD 2	3	6	23	13
		3	18	
		3	19	
HFD 3	2	10	26	24
		15	30	
HFD 4	6	3	17	232
		0.1	5	
		128	63	
		6	22	
		90	56	
		6	22	
HFD 5	8	23	36	208
		2	16	
		22	35	
		68	51	
		52	46	
		11	27	
		30	39	
		1	12	
HFD 6	3	7	24	38
		0.7	11	
		30	39	
HFD 7	8	12	28	308
		7	23	
		191	71	
		0.4	9	
		0.04	4	
		67	50	
		28	38	
		3	17	

Table S2. **Information about the genes selected for the obesity array.**

ENSDARG	GENE code ZFIN	NM	Target gene	Oligo sequence forward	Oligo sequence reverse
ENSDARG00000027740	ZDB-GENE-041010-89	NM_214715.2	adcyap1b	GATGCACTACAGCGCCTACT	ATCAGTGCCAAAAGCCAGGT
ENSDARG00000100086	ZDB-GENE-060825-220	NM_001045425.2	adipoqb	CCCCATAGAGAGCAGCATGTC	GCTCCTCGATTCCCTCAGTG
ENSDARG00000007490	ZDB-GENE-081022-145	NM_001128689.1	adrb1	TCATCGTATCTCTGGCGTGC	TGCAATTACGCACAGGGTCT
ENSDARG00000069089	ZDB-GENE-040817-1	XM_009303347.1	agrp	GGCTGGTTTTTGGTGAATGT	TTTCAAGGTGCTCCATTTCA
ENSDARG00000040298	ZDB-GENE-030131-1263	NM_001079861.1	apoa4b	AACTGCAGAGGAAACCGTCA	GCTCCCTCAGCACTTCAGTC
ENSDARG00000018817	ZDB-GENE-000412-1	NM_131595.2	bdnf	GGACACTTTCGAGCAGGTCA	CTCCAAAGGCACTTGGTTGC
ENSDARG00000036074	ZDB-GENE-020111-2	NM_131885.2	cebpa	GGAGCAAGCAAACCTCTACG	GATCTCGCTCAGGTCTCCAG
ENSDARG00000028661	ZDB-GENE-030616-82	NM_001098242.1	cntfr	ACCAGTGGCAACATGTGGAA	GACGTCGCTCTCAGTAGTGG
ENSDARG00000058285	ZDB-GENE-041010-9	NM_001005940.1	cpt1b	TATGACCGTTCAGACGCAGA	TACAGGCAGATGTGGCAGAG
ENSDARG00000038918	ZDB-GENE-070524-2	NM_001135976.2	drd1b	GGAAACACGTTGGTCTGTGC	CAAAGGCCACCCAAACATCG
ENSDARG00000020850	ZDB-GENE-990415-52	NM_131263	ef1a (eef1a1l1)	TTGAGAAGAAAATCGGTGGTGCTG	GGAACGGTGTGATTGAGGGAAATTC
ENSDARG00000010571	ZDB-GENE-041111-259	NM_001077279.1	ezh2	AAATCGGAGAAGGGTCCTGT	TCTGTTGGAGCTGAACATGC
ENSDARG00000017299	ZDB-GENE-040912-132	NM_001004682.1	fabp11a	TCAGAGCACCTTCAAAACCA	ACAAGTTTGCCGTTCTCGAT
ENSDARG00000099555	ZDB-GENE-061013-59	NM_001077257.2	foxo1	TGACGTGGAGTCGATCTTGC	GTGAGGTGGAATCTCAGCCG
ENSDARG00000013721	ZDB-GENE-050309-17	NM_001163806.1	g6pca.2	ATAGCCTATACTTGCGTGGCT	GGAAATGAGCGGCGATGAAG
ENSDARG00000043457	ZDB-GENE-030115-1	NM_001115114	gapdh	CGACTCCACCCATGGAAAGT	TAATGTTGGCTGGGTCCCTC
ENSDARG00000008840	ZDB-GENE-040426-1375	NM_201154	hmbs(a)	GTGTGTGGAATTGGACAACAAAGTG	CGAGGGCTGATGATGAGATATTGC
ENSDARG00000008884	ZDB-GENE-040426-1918	NM_212986	hprt1	CAGCGATGAGGAGCAAGGTTATG	GTCCATGATGAGCCCGTGAGG
ENSDARG00000099351	ZDB-GENE-021231-1	NM_173283.3	igfbp1a	AGGCCAAAGTCAACGCGATA	TTGTCAAGGGCTGTCTGGAG
ENSDARG00000035350	ZDB-GENE-980526-110	NM_131056.1	ins	CCCCAAGAGAGACGTTGAGC	CAGCACTGCTCTACAATGCC
ENSDARG00000011948	ZDB-GENE-020503-3	NM_001142672.1	insra	GATTCAGATGGCCGCAGAGA	AGACCCTTCCCACCCTTTCT
ENSDARG00000071524	ZDB-GENE-020503-4	NM_001123229.1	insrb	GGGCAGGGATCATTTGGGAT	CCATGACAACCAATGTCGGC
ENSDARG00000091085	ZDB-GENE-081001-1	NM_001128576	lepa	TTTTCCAGCTCTCCGCTCAAC	TGGGTTTGTCAGCGGGAAT
ENSDARG00000070961	ZDB-GENE-080104-1	NM_001309403	lepr	TTGGCTGGAATGGCAAATAC	GCTGAATCTCTGCCTGTGGT
ENSDARG00000087697	ZDB-GENE-990415-139	NM_131127.1	lpl	GGCCAAATTTGTCAACTGGT	CATGAGCGCCAAGACTGTAA
ENSDARG00000098439	ZDB-GENE-050410-7	NM_001017545	nr1h3 (lxra)	AGACCAGATCGCCCTACTCA	GCTGTCAATCGCTGGGTTAT
ENSDARG00000098439	ZDB-GENE-050410-7	NM_001017545.1	mc4r	CTGACCAACCGTGAGAGCAT	TGGTAGCGCAAAGCGTAGAA
ENSDARG00000015515	ZDB-GENE-021223-2	NM_173278.1	ncor1	AGGAGGAATCATGCACGCAA	TGCGGCTCTCTTTGGCTTTA
ENSDARG00000020482	ZDB-GENE-030131-713	NM_201579	nono	ATGGACAACACACCGATGCT	AAATCTCCGAAGCCTTGCCA
ENSDARG00000017180	ZDB-GENE-030131-3161	NM_001243875.1	npc1	CTGAGACCGTGGCCTTCTTT	CCATCCGATTGGCCTCTTGT
ENSDARG00000036222	ZDB-GENE-980526-438	NM_131074.2	npy	GTCTGCTTGGGGACTCTCAC	AGCGCTTGACCTTTTCCCAT
ENSDARG00000042824	ZDB-GENE-030723-2	NM_182889.1	nr3c1	CGTTTCCGCAAATGCCTCAT	GGAATGGACTGCTGCTGGAT
ENSDARG00000035285	ZDB-GENE-040426-993	NM_200276.2	nfe2l2a (nrf2)	CCATGTCATTCGATGAGTGC	TGAGATCAGTGGAAGGTGGA
ENSDARG00000098511	ZDB-GENE-010126-2	NM_001197161.2	ntrk2b	GCATCCACGTGTCGAGGTAT	CGACTGCAGGTCCATTTTGC
ENSDARG00000043135	ZDB-GENE-030513-2	NM_181438.3	pomc	ATGTTGTGTCCTGCTTGGCT	GGGTAGACGGGGGTTTCATC
ENSDARG00000031777	ZDB-GENE-041210-169	NM_001161333.1	pparaa	CGACGACACCTTCAACTCCT	GAGATCCGGATGAGTTCTCG
ENSDARG00000054323	ZDB-GENE-990415-211	NM_001102567.1	pparab	GTGACCTCGCACTGTTTGTC	CTCCTGCATTCGCTCGATA
ENSDARG00000044525	ZDB-GENE-990415-212	XM_005168286.2	pparda	ATCGACCTCTTCCTCAACGA	AAGCAGCCCGTCTTTATTCA
ENSDARG00000009473	ZDB-GENE-000112-47	NM_131468.2	ppardb	CGTCAACACAGCCTACCTGA	GCCACAGGGAGTCCATATCA
ENSDARG00000031848	ZDB-GENE-990415-213	NM_131467.1	pparg	TACGAGAACAACCCAACA	GTATCTGCTGTGCTCTGTA
ENSDARG00000057737	ZDB-GENE-070314-2	NM_001161551	rxraa	ACCCCACTCTCTCTCACCAA	GCAGCTGTACACACCGTAGT
ENSDARG00000035819	ZDB-GENE-070112-1762	NM_001080174	sirt3	TCAGCACACCAAGTGGAATC	CGTCAGGTTGGGTTGGTAAT
ENSDARG00000035559	ZDB-GENE-990415-270	NM_131327.2	tp53	GGGCAATCAGCGAGCAAA	ACTGACCTTCCTGAGTCTCCA
ENSDARG00000023151	ZDB-GENE-010503-1	NM_199523.2	ucp1	GGCCTCTACGACAACGTCAA	TTGTATCGTCTGCCCACACC
ENSDARG00000040925	ZDB-GENE-980526-524	NM_178219.2	wnt10b	CCGGATGAGGTCACAATGCT	TGGCACTTGCACTTTCTCCT
ENSDARG00000102464	ZDB-GENE-980526-87	NM_130937.1	wnt5b	CCCTCATCGTCTGCAACTCA	TCTGACCCTGAGATAGGCCC

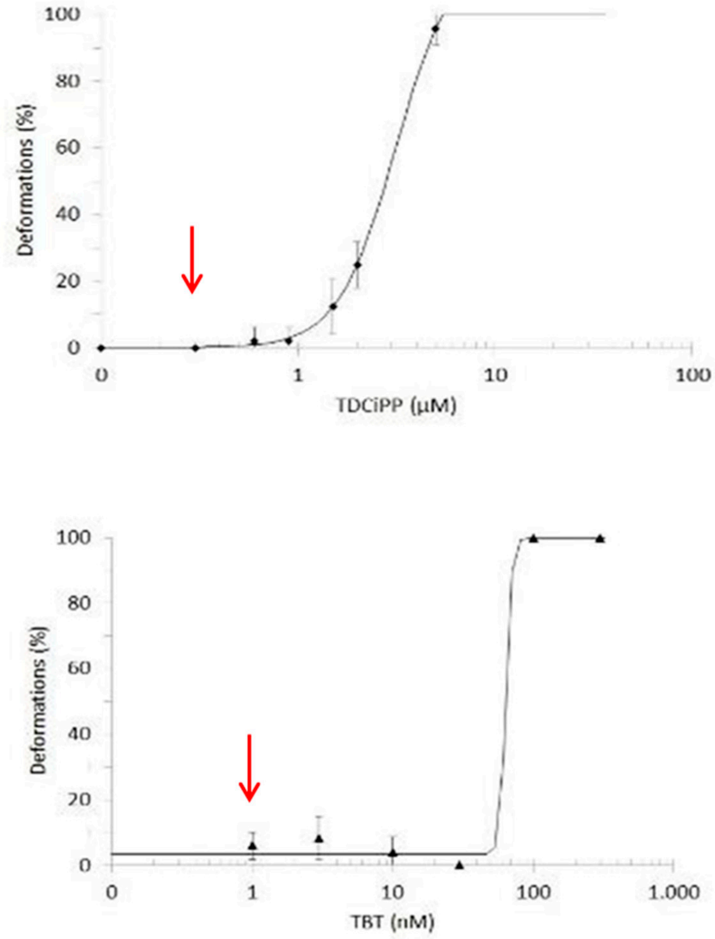

Figure S2. Dose-response curves for selected environmental chemicals, showing percentage of deformed larvae after exposure to (**a**) TDCiPP, (**b**) TBT. The red arrows indicate the non-toxic concentrations selected for this study.

Table S3. Overview of all adipocyte measurements of fish exposed to different environmental chemicals. All images made with SRS imaging can be found in the supplementary materials.

	**average number of adipocytes**	**average volume per fish in pL**
solvent control	3.0	229
TDCiPP	2.5	25
TBT	4.6	164

**FISH ID**	**number of adipocytes**	**adipocyte volume in pL**	**approximate diameter in µm**	**total volume per fish in pL**
solvent 1	1	80	53	80
solvent 2	1	209	74	209
solvent 3	1	90	56	90
solvent 4	3	58	48	160
		100	58	
		1	13	
solvent 5	3	3	17	46
		21	34	
		23	35	
solvent 6	4	71	51	1235
		70	51	
		137	64	
		958	122	
solvent 7	1	4	19	4
solvent 8	10	0.1	6	12
		1	13	
		3	17	
		0.1	6	
		1	14	
		0.3	8	
		0.1	5	
		6	22	
		0.1	5	
		1	12	
TDCiPP 1	1	26	37	26
TDCiPP 2	4	0.1	5	16
		8	25	
		6	22	
		2	17	
TDCiPP 3	1	12	29	12
TDCiPP 4	4	0.6	10	13
		2	16	
		0.03	4	
		10	27	
TDCiPP 5	1	7	24	7
TDCiPP 6	6	3	19	34
		1	14	
		3	17	
		3	18	
		16	31	
		7	24	
TDCiPP 7	1	1	12	1
TDCiPP 8	2	78	53	91
		13	29	
TBT 1	2	177	70	216
		39	42	
TBT 2	6	169	69	550
		280	81	

		39	42	
		43	43	
		11	28	
		7	24	
TBT 3	3	1	12	8
		5	21	
		2	16	
TBT 4	3	61	49	387
		214	74	
		112	60	
TBT 5	14	12	28	61
		0.2	8	
		5	21	
		1	13	
		26	37	
		0.3	9	
		0.4	9	
		0.4	9	
		0.6	10	
		11	28	
		1	13	
		0.05	4	
		0.5	10	
		2	15	
TBT 6	5	0.2	7	28
		0.1	6	
		1	13	
		2	17	
		25	36	
TBT 7	2	0.5	10	48
		47	45	
TBT 8	2	3	18	15
		12	28	
